# Effectiveness of the Viet Nam Produced, Mouse Brain-Derived, Inactivated Japanese Encephalitis Vaccine in Northern Viet Nam

**DOI:** 10.1371/journal.pntd.0001952

**Published:** 2012-12-13

**Authors:** Florian Marks, Thi Thu Yen Nguyen, Nhu Duong Tran, Minh Hong Nguyen, Hai Ha Vu, Christian G. Meyer, Young Ae You, Frank Konings, Wei Liu, Thomas F. Wierzba, Zhi-Yi Xu

**Affiliations:** 1 International Vaccine Institute, Seoul, South Korea; 2 National Institute of Hygiene and Epidemiology, Ha Noi, Viet Nam; 3 Bernhard Nocht Institute for Tropical Medicine, Hamburg, Germany; 4 Harvard School of Public Health, Boston, Massachusetts, United States of America; Centre for Cellular and Molecular Biology (CCMB), India

## Abstract

**Background:**

Japanese encephalitis (JE) is a flaviviral disease of public health concern in many parts of Asia. JE often occurs in large epidemics, has a high case-fatality ratio and, among survivors, frequently causes persistent neurological sequelae and mental disabilities. In 1997, the Vietnamese government initiated immunization campaigns targeting all children aged 1–5 years. Three doses of a locally-produced, mouse brain-derived, inactivated JE vaccine (MBV) were given. This study aims at evaluating the effectiveness of Viet Nam's MBV.

**Methodology:**

A matched case-control study was conducted in Northern Viet Nam. Cases were identified through an ongoing hospital-based surveillance. Each case was matched to four healthy controls for age, gender, and neighborhood. The vaccination history was ascertained through JE immunization logbooks maintained at local health centers.

**Principal Findings:**

Thirty cases and 120 controls were enrolled. The effectiveness of the JE vaccine was 92.9% [95% CI: 66.6–98.5]. Confounding effects of other risk variables were not observed.

**Conclusions:**

Our results strongly suggest that the locally-produced JE-MBV given to 1–5 years old Vietnamese children was efficacious.

## Author Summary

Japanese encephalitis (JE) is a disease caused by a flavivirus transmitted by mosquitoes. Although pigs and wild birds are main reservoirs of the disease, it is occasionally transmitted to humans. The majority of infections in humans are asymptomatic. In persons developing encephalitis, JE has a high case-fatality rate and, among survivors, JE frequently causes persistent neurological sequelae and mental disabilities. Therefore, it is a public health concern in many parts of Asia and many countries vaccinate against JE. Since 1997, children in Vietnam are vaccinated in high risk areas and receive a locally-produced vaccine. This study is aimed at evaluating the effectiveness of the Vietnamese JE vaccine through a case-control study, in which 30 cases and 120 controls were enrolled. The effectiveness of the JE vaccine was 92.9% [95% CI: 66.6–98.5], which suggests that the locally-produced JE vaccine given to 1–5 year old Vietnamese children was efficacious.

## Introduction

Japanese encephalitis (JE) is a mosquito-borne flaviviral disease endemic in many regions of Asia [1]. *Culex tritaeniorhynchus*, the principal mosquito vector of the JE virus (JEV), preferentially breeds in rice fields [2], [3]. Swine are potent amplifiers of the virus and exhibit rapidly after virus transmission considerable viral loads. Thus, *Culex* mosquitoes breeding in rice fields and feeding on swine, are critical ecological factors favoring JE transmission to humans in rural areas. Prior to the availability and introduction of vaccines, JE was a significant cause of mortality in the northern provinces of Viet Nam with an annual incidence of 5–15/100,000 [4].

Most JE infections (96%–99.9%) are asymptomatic or present as a mild disease only with rather non-specific flu-like symptoms. However, among symptomatic patients who exhibit symptoms of encephalitis and/or serious neurologic infection, the case-fatality ratio can be as high as 10%–30% [5], [6]. Among survivors, 30% to 50% of individuals suffer from chronic, severe neuropsychiatric disabilities [5]–[7]. Why only a small proportion of infected individuals experience severe disease is not clear. Reasons may include host genetic factors, but also virulence factors of differing virus strains. Children younger than 15 years are at the highest risk of infection and the incidence peaks at three to ten years of age [7]. JE infections efficiently induce protective immunity [8] and seroprevalence studies indicate almost universal exposure to the infection in endemic areas by adulthood [9].

Specific antiviral treatment for JE is not available [10] and care of patients strongly depends on supportive measures. Vaccination is the primary strategy for prevention of infection [1] and has been shown to dramatically reduce the disease incidence in South Korea [11], [12], Japan [13], China [14], [15], Thailand [15] and Taiwan [16]. In Viet Nam, children receive three pediatric doses (0.5 ml/dose) of a locally-produced, mouse brain-derived, inactivated JE vaccine (MBV; Nakayama strain; VaBiotech, National Institute for Hygiene and Epidemiology (NIHE), Ha Noi, Viet Nam) with the first two doses at one year of age given at an interval of two weeks followed by a third, booster dose one year later. The production of the MBV in Viet Nam was initiated in 1989 and supported by technology transfer from Japan. Bridging studies suggest that the Vietnamese MBV has an immunogenicity similar to that of the Japanese vaccine, reaching nearly 100% immunogenicity in children after the application of two doses [1]. The vaccine was integrated into Viet Nam's national Extended Program of Immunizations (EPI) in selected districts of Ha Tay and Hai Phong provinces in 1997.

Hospital-based surveillance of all patients presenting with an acute encephalitis syndrome (AES) is ongoing in the Ha Tay and Hai Phong Provincial Hospitals in the North of Viet Nam. Two provincial hospitals in the Ha Tay Province as well as the National Pediatric Hospital in Ha Noi are used as referral hospitals for JE surveillance since January 1, 2004. These hospitals jointly account for approximately 95% of all cases of acute encephalitis notified in the Ha Tay Province. In Hai Phong province, cases are identified through the national AES surveillance system. Cerebrospinal fluid (CSF) is collected at admission. The presence of immunglobulin-M (IgM) antibodies to JEV (anti-JEV) in CSF is defined as one of the criteria for a JE diagnosis [17]–[19]. Initial testing of specimens is performed at the National Pediatric Hospital Laboratory and Laboratory of Ha Tay Preventive Medicine Center, and confirmatory testing of all specimens was done by sending coded samples to the Department of Virology, Armed Forces Research Institute of Medical Sciences (AFRIMS), Bangkok, Thailand.

The objective of the present study was to assess in a case-control design the effectiveness of this MBV JE vaccine.

## Methods

### Ethical clearance

The study was conducted according to ethical principles consistent with the International Guideline for Ethical Reviews of Epidemiologic Studies [20]. The Institutional Review Board of the International Vaccine Institute, Seoul, Republic of Korea, and the local ethical committee of NIHE, Ha Noi, Viet Nam approved the study and granted ethical clearance. Vaccination status' of participants were assessed from vaccination records of the health centers. During visits of the households of children that were aimed at assessing the distances of piggeries and ricefields from the houses, oral informed consent was obtained from parents/guardians of cases and controls and documented in the questionnaire. Oral consent was considered appropriate for the study and approved by both Ethical Review Boards.

### Study design

For this matched case-control study, patients with a confirmed diagnosis of JE and younger than 15 years of age were identified from the database of the surveillance hospitals from January 2004 to December 2007. Older children were not recruited, as they could not have received JE vaccines through the national immunization program.

Controls were chosen from the birth registry of all births in the health center of the village of the case and matched for gender, age (+/− six months), and proximity of their house.

Only after four controls had been selected from birth registry and agreed upon by the study team, the vaccination record books were opened and the individual vaccination status was assessed. JE vaccination is provided during an annual mass campaign and vaccination records are kept at the health centers.

The houses of cases and controls were visited and as an indicator of potential exposure to infection and the distances to piggeries and rice fields were assessed by the study team after obtaining oral informed consent from the adult/guardian present.

### Statistics

A Student's t-test for unequal distributions was used to compare the age distribution of cases and controls. A chi-square test was employed to compare cases and controls for the proportion of males, proportion living within ≤50 meters of a piggery and percent living ≤30 meters of rice fields. A matched conditional logistic regression model was employed to evaluate the protective effect of immunization. A single dependent (JE disease) and independent variable (immunization status) was entered into the model. Odds ratios (OR), 95% confidence intervals (CI), and *p* values were calculated from model parameters. The protective effect of immunization was assessed for residents receiving three or more doses relative to two or less doses. Vaccine effectiveness (VE) was calculated as VE = 1−OR×100 [21]. The threshold of statistical significance was *p*<0.05. All statistical analyses were performed using the Stata v 10.0 software (Stata Corp., Texas, USA).

## Results

We identified 30 laboratory-confirmed cases of JE infection and these were matched to 120 controls. After selection of controls was completed, it became evident that two controls were chosen who had no information on their immunization status in the birth registries. These two controls were excluded from the calculation of vaccine effectiveness. As cases and controls were matched for age and gender, no significant differences were observed between cases and controls for these variables. Cases and controls resided equidistantly from rice fields and piggeries (Table 1).

**Table 1:** Comparison of socio-economic variables for cases and controls.

	Cases (n = 30)	Controls (n = 120)	*P*
Male	.63*	.63*	1.00‡
Mean age, years (Range)	6.8 (4–13)	6.9 (3–13)	0.63†
Rice fields ≤50 m	.37*	.37*	1.00‡
Piggeries ≤30 m	.6*	.58*	0.83‡

‡ chi-square test;

† , paired t-test for unequal distribution,

* proportions.

Only individuals who had received the full course (three doses) of JE vaccine were considered as vaccinated; two cases and two controls received one and two cases and six controls two doses of JE vaccine, respectively, and were considered as not vaccinated (Table 2).

**Table 2:** Vaccine effectiveness 1∶4 case control (≥3 doses = vaccinated).

		Cases	Controls*
Vaccinated	Yes	18	102
	No	12	16

Vaccine effectiveness is 92.9% and corresponding 95% confidence intervals are (66.6%, 98.5%).

* Vaccination status unknown for two controls (excluded from calculation).

Among the 30 laboratory-confirmed cases, the proportion of cases vaccinated was 60.0%, compared to 86.4% among the 118 control individuals. Based on the matched analysis vaccine effectiveness reached 92.9% [95% CI: 66.6–98.5%] (Table 2).

## Discussion

Three previous trials have evaluated the effectiveness of the JE MBV. A randomized double-blinded study conducted in northern Thailand, using JE MBV produced in Thailand, yielded an overall effectiveness of 91% [95% CI: 70.0–97.0] [22]. Another trial in Taiwan evaluated a Taiwanese vaccine and revealed an effectiveness of approximately 85% when two or more doses were administered [23]. A case-control study in Thailand showed an effectiveness of 94.6% in children ≥18 years of age [24]. The present matched case-control study suggests that the MBV produced in Viet Nam yields results that are similar to those of the Thailand and Taiwan studies.

Even though the MBV has an excellent efficacy, its usage was recently restricted after considerable safety concerns were raised. Severe reactions such as hypersensitivity, including generalized urticaria and angioedema, occurred at far higher rates than observed in other routine vaccinations [25], [26], [27], [28]. Consequently, Japan no longer recommends JE MBV vaccination [29], [30] and recently introduced a Vero cell-derived JE vaccine; other countries are currently in the process of phasing out the MBV (e.g., Thailand, Sri Lanka). Nevertheless, MBV safety issues have not led to any restrictions in its use in Viet Nam. However, Viet Nam is currently developing its own Vero cell-derived vaccine, which is foreseen to be available for use in 2014/2015.

The Vietnamese government has announced that it would eliminate clinical JE by 2015. However, in contrast to poliomyelitis virus, for which humans are the only hosts, the JE virus is enzootic and is, therefore, unlikely to be entirely eradicated from the environment. A recent JE surveillance study has shown that, even in the virtual absence of manifest human disease after vaccination, JEV is still widespread among swine [31], showing that active transmission is perpetuated and that protective immunity of humans through persistent vaccination is a key measure to preventing disease in humans [13]. Therefore, controlling clinical JE disease through vaccination would not impact on reducing or eradicating the circulation of the virus within the vectors and animal hosts as JE is not transmitted from person to person and JE vaccination does probably not confer herd immunity [32].

Depending on the potency of enzootic transmission and the age-specific risks of natural human infection, the age for primary vaccination differs between countries. Most countries give one to two booster doses after the initial three-dose regimen [1].

In contrast to Japan and Korea, where nation-wide changes in lifestyle have provided additional contributions to the control of JE [11], [33], rural areas in Viet Nam have so far largely remained agrarian. Rice paddies cover extensive geographic areas, and little changes only have occurred in the natural environment and living conditions. Even though the effectiveness of the vaccination program appears to be high, an annual average incidence of 3.4/100,000 was observed among children less than 10 years of age. The current JE immunization program may be improved by immunizing younger children (6–23 months of age) who are at the highest risk of patent infection, and providing a booster dose at 3–5 years after initial immunization with the three-dose regimen, reducing waning immunity in immunized children [1].

An inherent problem of inactivated vaccines is that due to their low immunogenicity, multiple vaccinations are required in order to induce and maintain sustained levels of protective immunity. Following the initial three doses given at immunization, the effectiveness of the MBV declines over years [34]. Therefore, the introduction of one or more booster doses using the adult formulation to children at school age is recommended to ensure protective immunity against JE using MBV.

## Supporting Information

Checklist S1 STROBE Checklist.
(DOC)
