# Supplementary material for: Effectiveness of the Viet Nam Produced, Mouse Brain-Derived, Inactivated Japanese Encephalitis Vaccine in Northern Viet Nam
Source: PLoS Negl Trop Dis. 2012 Dec 13;6(12):e1952. doi: 10.1371/journal.pntd.0001952 (PMC3521705; doi:10.1371/journal.pntd.0001952)
Supplement: Checklist S1 — STROBE Checklist. (DOC) [file pntd.0001952.s001.doc]

STROBE Statement—Checklist of items that should be included in reports of ***case-control studies***

|  | Item No | Recommendation |
| --- | --- | --- |
| **Title and abstract**  **DONE** | 1 | (*a*) Indicate the study’s design with a commonly used term in the title or the abstract  **DONE – in abstract** |
|  |  | (*b*) Provide in the abstract an informative and balanced summary of what was done and what was found **DONE – in abstract** |
| Introduction | | |
| Background/rationale  **DONE** | 2 | Explain the scientific background and rationale for the investigation being reported **DONE - introduction** |
| Objectives  **DONE** | 3 | State specific objectives, including any prespecified hypotheses **DONE – last sentence of introduction** |
| Methods | | |
| Study design  **DONE** | 4 | Present key elements of study design early in the paper **DONE – method section** |
| Setting  **DONE** | 5 | Describe the setting, locations, and relevant dates, including periods of recruitment, exposure, follow-up, and data collection **DONE – introduction and method section** |
| Participants  **DONE** | 6 | (*a*) Give the eligibility criteria, and the sources and methods of case ascertainment and control selection. Give the rationale for the choice of cases and controls **DONE – method section** |
|  |  | (*b*) For matched studies, give matching criteria and the number of controls per case **DONE – method section** |
| Variables  **DONE** | 7 | Clearly define all outcomes, exposures, predictors, potential confounders, and effect modifiers. Give diagnostic criteria, if applicable **DONE – method section** |
| Data sources/ measurement  **DONE** | 8* | For each variable of interest, give sources of data and details of methods of assessment (measurement). Describe comparability of assessment methods if there is more than one group **DONE – method section** |
| Bias  **DONE** | 9 | Describe any efforts to address potential sources of bias **DONE – method section** |
| Study size  **DONE** | 10 | Explain how the study size was arrived at **DONE – method section** |
| Quantitative variables  **DONE** | 11 | Explain how quantitative variables were handled in the analyses. If applicable, describe which groupings were chosen and why **DONE – method section** |
| Statistical methods  **DONE** | 12 | (*a*) Describe all statistical methods, including those used to control for confounding  **DONE – method section** |
|  |  | (*b*) Describe any methods used to examine subgroups and interactions **N/A** |
|  |  | (*c*) Explain how missing data were addressed **DONE – method section** |
|  |  | (*d*) If applicable, explain how matching of cases and controls was addressed |
|  |  | (*e*) Describe any sensitivity analyses **N/A** |
| Results | | |
| Participants  **DONE** | 13* | (a) Report numbers of individuals at each stage of study—eg numbers potentially eligible, examined for eligibility, confirmed eligible, included in the study, completing follow-up, and analysed **DONE – result section** |
|  |  | (b) Give reasons for non-participation at each stage **N/A** |
|  |  | (c) Consider use of a flow diagram **N/A** |
| Descriptive data  **DONE** | 14* | (a) Give characteristics of study participants (eg demographic, clinical, social) and information on exposures and potential confounders **DONE – result section** |
|  |  | (b) Indicate number of participants with missing data for each variable of interest **DONE – result section** |
| Outcome data  **DONE** | 15* | Report numbers in each exposure category, or summary measures of exposure **DONE – result section** |
| Main results  **DONE** | 16 | (*a*) Give unadjusted estimates and, if applicable, confounder-adjusted estimates and their precision (eg, 95% confidence interval). Make clear which confounders were adjusted for and why they were included **DONE – result section** |
|  |  | (*b*) Report category boundaries when continuous variables were categorized **N/A** |
|  |  | (*c*) If relevant, consider translating estimates of relative risk into absolute risk for a meaningful time period **N/A** |

| Other analyses | 17 | Report other analyses done—eg analyses of subgroups and interactions, and sensitivity analyses |
| --- | --- | --- |
| Discussion | | |
| Key results | 18 | Summarise key results with reference to study objectives |
| Limitations | 19 | Discuss limitations of the study, taking into account sources of potential bias or imprecision. Discuss both direction and magnitude of any potential bias |
| Interpretation | 20 | Give a cautious overall interpretation of results considering objectives, limitations, multiplicity of analyses, results from similar studies, and other relevant evidence |
| Generalisability | 21 | Discuss the generalisability (external validity) of the study results |
| Other information | | |
| Funding | 22 | Give the source of funding and the role of the funders for the present study and, if applicable, for the original study on which the present article is based |

*Give information separately for cases and controls.

**Note:** An Explanation and Elaboration article discusses each checklist item and gives methodological background and published examples of transparent reporting. The STROBE checklist is best used in conjunction with this article (freely available on the Web sites of PLoS Medicine at http://www.plosmedicine.org/, Annals of Internal Medicine at http://www.annals.org/, and Epidemiology at http://www.epidem.com/). Information on the STROBE Initiative is available at http://www.strobe-statement.org.
